# Diabetes Is Associated with Worse Postoperative Mortality and Morbidity in Bariatric Surgery, Regardless of the Procedure

**DOI:** 10.3390/jcm13113174

**Published:** 2024-05-28

**Authors:** Omar Khalil, Soha Dargham, Amin Jayyousi, Jassim Al Suwaidi, Charbel Abi Khalil

**Affiliations:** 1 Research Department, Weill Cornell Medicine—Qatar, Doha P.O. Box 24144, Qatar; 2 Department of Medicine, Virginia Commonwealth University Health, Richmond, VA 23298, USA; 3 Department of Medical Education, Weill Cornell Medicine—Qatar, Doha P.O. Box 24144, Qatar; 4 Department of Endocrinology, Hamad Medical Corporation, Doha P.O. Box 3050, Qatar; 5 Heart Hospital, Hamad Medical Corporation, Doha P.O. Box 3050, Qatar; jalsuwaidi@hamad.qa; 6 Sanford and I. Weill Department of Medicine, Weill Cornell Medicine, New York, NY 10065, USA

**Keywords:** obesity, diabetes, bariatric surgery, mortality, sleeve gastrectomy, Roux-en-Y, gastric banding

## Abstract

**Background/Objectives**: Bariatric surgery is a central cornerstone in obesity treatment. We aimed to assess the impact of diabetes on the postoperative outcomes of bariatric surgery and compare three techniques: sleeve gastrectomy, Roux-en-Y, and gastric banding. **Methods**: We extracted data from the National Inpatient Sample (2015–2019) using ICD codes. The primary outcome was postoperative mortality. Secondary outcomes were major bleeding, atrial fibrillation, and acute renal failure. **Results**: Among patients who underwent sleeve gastrectomy, diabetes was associated with a higher adjusted risk of mortality (aOR 2.07 [1.36–3.16]), atrial fibrillation, and acute renal failure, but a similar risk of bleeding. Among patients who underwent Roux-en-Y, diabetes did not increase mortality and bleeding risk. Still, it was associated with a higher risk of atrial fibrillation and acute renal failure. Among patients who underwent gastric banding, diabetes was only associated with a higher risk of bleeding. When comparing the three techniques in diabetes patients, Roux-en-Y was significantly associated with higher mortality and acute renal failure risk when compared to the other procedures. Bleeding was more common in Roux-en-Y than in Sleeve. **Conclusions**: In total, diabetes is associated with worse postoperative outcomes in bariatric surgery, regardless of the technique. Among diabetes patients, Roux-en-Y was associated with the highest mortality and morbidity.

## 1. Introduction

Obesity has reached epidemic proportions around the world [1]. As the prevalence and incidence of obesity continue to increase, bariatric surgery has become one of the main cornerstones of the treatment [2]. Candidates who may benefit from the surgery include adults with a BMI of ≥35 kg/m^2^ and those with a BMI between 30 and 35 kg/m^2^ with metabolic disease [3].

The prevalence and incidence of type 2 diabetes (T2D) are also on the rise. Despite a recent temporal decrease in diabetes-related mortality [4], patients with diabetes still present an excess risk of developing cardiovascular complications and dying earlier than their non-diabetes counterparts within the same age and sex category [5]. These complications stem from elevated blood glucose levels over time, culminating in the thickening and narrowing of blood vessels, predisposing individuals to arterial blockages and impaired blood flow [6,7]. Conversely, microvascular complications manifest predominantly in small blood vessels, predominantly causing diabetic retinopathy, diabetic nephropathy, and diabetic neuropathy [8]. Chronic hyperglycemia triggers a cascade of events, including oxidative stress and inflammation, fostering endothelial dysfunction and aberrant angiogenesis, ultimately leading to tissue damage and organ dysfunction [9].

Because of its close association with obesity, bariatric surgery is recommended in diabetes patients with obesity [10]. Bariatric procedures often lead to alterations in gut hormones, such as increased glucagon-like peptide 1 (GLP-1) and peptide YY (PYY), which enhance insulin secretion, improve insulin sensitivity, and suppress appetite. The long-term incidence of new-onset diabetes is drastically reduced in non-diabetic bariatric patients who undergo bariatric surgery [11]. Further, research has shown a significant reduction in glycemia and decreased reliance on diabetes medications following bariatric surgery in patients with diabetes [12]. Many individuals experience partial or complete remission of T2D, often within days to weeks post-surgery before substantial weight loss occurs. Moreover, bariatric surgery has been shown to mitigate other cardiometabolic risk factors associated with diabetes, such as hypertension and dyslipidemia, thereby conferring additional health benefits [13]. 

Diabetes is a known risk factor for numerous intra- and postoperative complications, including infections, cardiac events, and cerebrovascular accidents, and is known to increase overall postoperative mortality [14]. This study aimed to determine the impact of diabetes on postoperative mortality and morbidity in sleeve gastrectomy, Roux-en-Y, and gastric banding bariatric surgeries. Further, we compared those three procedures within the diabetes population.

## 2. Materials and Methods

### 2.1. Data Source and Study Population

Data was obtained from the National Inpatient Sample (NIS), a large American administrative database containing health information on millions of patients across over a thousand hospitals in 44 states [15]. This information includes patient characteristics, medical diagnoses, and healthcare costs and outcomes, which are categorized using the International Classification of Diseases (ICD) coding system. The ninth edition (ICD-9) was used until 30 September 2015, and the 10th edition (ICD-10) starting October 1 of that year [15]. Each admission consists of unique identifiers in the database, preventing repeat admissions for the same patient.

### 2.2. Study Population and Outcomes

Adult patients (>18 years of age and older) whose primary diagnosis was bariatric surgery were included in this analysis. Bariatric surgery consisted of Roux-en-Y bypass [ICD-9: 44.31, 44.38, 43.39; ICD-10: Z90.3, Z98.0, Z98.84], gastric banding [ICD-9: 44.95; ICD-10: 0DV64CZ], and sleeve gastrectomy [ICD-9: 43.82; ICD-10: 0DB64Z3]. Patients were stratified according to type 2 diabetes (ICD-9: 250. x, ICD-10: E11.9) and analyzed accordingly. The primary outcome was postoperative in-hospital mortality. Secondary outcomes were major postoperative major bleeding, atrial fibrillation, and acute renal failure. Patients with missing outcomes, age, and sex were excluded from inclusion in the analysis pool. 

### 2.3. Analysis Plan and Statistics

The trend of diabetes cases in each bariatric surgery procedure was first analyzed, followed by comparing diabetes patients to non-diabetes ones. Finally, the three techniques were compared in all patients with diabetes. Patients’ characteristics and outcomes are presented as frequency distribution, mean (standard deviation), median (interquartile range), and odds ratios (95% confidence interval) as appropriate. A simple linear regression assessed the trend across the observational period. Using logistic regression, ORs were adjusted for confounding factors of demographics and comorbidities that differed across the two compared groups. Those factors included age, sex, race, income, hypertension, smoking, dyslipidemia, peripheral vascular disease, valvular disease, renal failure, and coronary artery disease. To estimate the nationwide data and outcomes, a weight (DISCWT) was used to estimate the nationwide data per the recommendation of the healthcare cost and utilization project (HCUP) to which the NIS belongs [16]. A *p*-value < 0.05 was considered statistically significant. We organized, filtered, and analyzed the data using the statistical computer software SPSS (IBM, Armonk, NY, USA, version 26.0).

## 3. Results

### 3.1. Study Group

**Figure 1:**
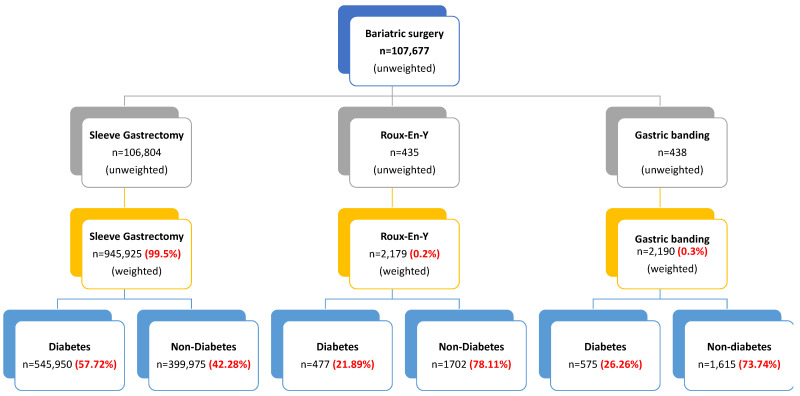
Flow chart of the study.

The unweighted study group obtained from the NIS database consisted of 107,677 patients who underwent either sleeve gastrectomy (106,804 patients), Roux-en-Y (435 patients), or gastric banding (438 patients) between 2015 and 2019 (Figure 1). After excluding missing and incomplete records and weighting the data, the total number of patients was 948,575. Interestingly, most patients (99.5%) had a sleeve gastrectomy, and 0.2% and 0.3% had Roux-en-Y and gastric banding, respectively. Diabetes represented more than 50% in the sleeve gastrectomy group, 21.89% in the Roux-en-Y group, and 26.6% in the gastric banding group.

**Figure 2:**
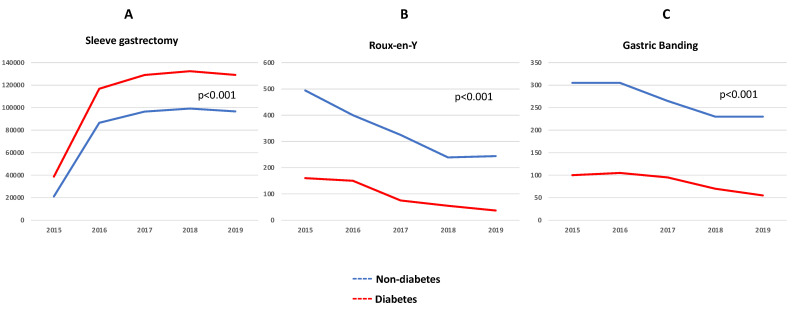
Temporal trend of the total number of patients who were hospitalized for sleeve gastrectomy (**A**), Roux-en-Y-y (**B**), and gastric banding (**C**). Patients were further stratified into non-diabetes (blue line) and diabetes (red line). The *X*-axis represents years. The *Y*-axis represents the total number of patients.

The trend in bariatric surgery over the years 2015–2019 supports a leaning towards sleeve gastrectomy compared to Roux-en-Y and gastric banding. The number of patients undergoing sleeve gastrectomy grew significantly by almost 3.5-fold during the observation period (*p* < 0.001) (Figure 2A), which was paralleled with a decrease in the number of patients who underwent Roux-en-Y (Figure 2B) and gastric banding (Figure 2C) (*p* < 0.001 for all).

### 3.2. Comparison of Patients with and without Diabetes

#### 3.2.1. Sleeve Gastrectomy

Diabetes patients were older and more likely to have a lower income than non-diabetes ones (49 [12] vs. 42 [12], respectively, *p* < 0.001). Both groups had more females than males (Supplementary Table S1). However, a higher proportion of males is observed in patients with diabetes (28.5% vs. 18%, diabetes vs. non-diabetes, *p* < 0.001). Diabetes patients had a higher prevalence of cardiovascular risk factors such as smoking (23% vs. 18.3%), hypertension (56.6% vs. 33.4%), and dyslipidemia (44.8% vs. 20.1%) (diabetes vs. non-diabetes, respectively, *p* < 0.001 for all). The results in Table 1 indicate no significant difference between diabetes and non-diabetes patients regarding major bleeding events. On the other hand, the risk of mortality was almost doubled in the presence of diabetes (aOR = 2.07 [1.36–3.16]). Additionally, atrial fibrillation and acute renal failure were more likely to occur in patients with diabetes (aOR = 1.07 [1.02–1.11], 1.51 [1.39–1.64], respectively). 

#### 3.2.2. Roux-en-Y

Diabetes patients were older (53 [12] vs. 45 [13]), more likely to be males (31.3% vs. 24.6%) and had a higher income (28.18% vs. 22.05%) (diabetes vs. non-diabetes, respectively, *p* < 0.001 for all) (Supplementary Table S3). None of the patients had CAD in either group. Five deaths occurred in patients with non-diabetes compared to none in diabetes. However, the bleeding risk was almost threefold in the presence of diabetes (aOR 2.94 [1.42–5.88]) (Table 2).

#### 3.2.3. Gastric Banding

Diabetes patients were older (53 [12] vs. 45 [13]), more likely to be males (31.3% vs. 24.6%) and had a higher income (28.18% vs. 22.05%) (diabetes vs. non-diabetes, respectively, *p* < 0.001 for all) (Supplementary Table S3). None of the patients had CAD in either group. Five deaths occurred in patients with non-diabetes compared to none in diabetes. However, the bleeding risk was almost threefold in the presence of diabetes (aOR 2.94 [1.42–5.88]) (Table 3).

### 3.3. Comparison of Bariatric Surgery Outcomes among Patients with Diabetes

#### 3.3.1. Sleeve Gastrectomy vs. Roux-en-Y among Patients with Diabetes

There was no significant difference between sleeve gastrectomy and Roux-en-Y regarding bleeding events (Table 4). However, there was a significant difference between the two surgeries regarding mortality, atrial fibrillation, and acute renal failure. More specifically, diabetes patients undergoing Roux-en-Y were more likely to die (aOR 8.94 [5.53–16.49]) and develop acute renal failure (aOR 3.68 [2.92–4.63]) and atrial fibrillation (aOR 1.62 [1.48–1.81]).

#### 3.3.2. Sleeve Gastrectomy vs. Gastric Banding among Patients with Diabetes

The results in Supplementary Table S4 indicate a significant difference between the two procedures regarding bleeding, atrial fibrillation, and acute renal failure. Patients undergoing gastric banding had a higher risk of bleeding and acute renal failure (aOR 7.1 [5.79–9.05]), 3.89 [2.9–5.2]; respectively) but were less likely to develop atrial fibrillation than their counterparts with diabetes undergoing sleeve gastrectomy (aOR 0.58 [0.41–0.83]). Seventy-five deaths occurred in the sleeve patients but none in the gastric banding ones.

#### 3.3.3. Roux-en-Y versus Gastric Banding among Patients with Diabetes

As seen in Supplementary Table S5, there was no significant difference between Roux-en-Y and gastric banding in terms of atrial fibrillation. On the other hand, the bleeding risk has majored almost 11-fold in the gastric banding group. The risk of acute renal failure was lower (aOR 0.34 [0.22–0.53]). Five deaths occurred in the Roux-en-Y group, but none in the gastric banding ones.

## 4. Discussion

This analysis of the NIS database shows that diabetes patients had generally worse postoperative outcomes than non-diabetes ones, regardless of the type of bariatric surgery performed. Further, Roux-en-Y is associated with a higher risk of mortality and acute renal failure than sleeve gastrectomy but lower bleeding risk than gastric banding. Finally, sleeve gastrectomy is associated with lower bleeding and acute renal failure than gastric banding.

Bariatric surgeries work via a restrictive or malabsorptive mechanism [17,18]. The gastric band operation and sleeve gastrectomy are restrictive procedures. The Roux-en-Y surgery has a restrictive and malabsorptive mechanism. Sleeve gastrectomy involves irreversible laparoscopic removal of a large stomach section along the greater curvature to end up with a small gastric tube and pouch of about 50–150 mL capacity [19]. The gastric fundus is also removed in sleeve gastrectomy, which decreases ghrelin production, thus reducing appetite and food intake, leading to weight loss [20]. Gastric banding is a laparoscopic procedure in which the upper section of the stomach is tied with an adjustable gastric band that reduces the gastric pouch size to 15–30 mL, thus reducing food intake and causing weight loss [19,21]. Lastly, Roux-en-Y is a laparoscopic procedure in which a small gastric pouch of less than 30 mL is made using the upper portion of the stomach and then connected to the jejunum [19,22]. This bypasses the rest of the stomach and duodenum, reducing food absorption while decreasing appetite and increasing insulin sensitivity, leading to weight loss and better diabetes control.

Roux-en-Y has historically been the most performed surgery due to its restrictive and malabsorptive mechanism, which is thought to optimize weight loss [19]. However, sleeve gastrectomy use has increased recently due to its relatively safer complication profile than other bariatric surgeries such as Roux-en-Y and gastric banding [23]. The literature needs more data on complication differences between Roux-en-Y, gastric banding, and sleeve gastrectomy, with discrepancies sometimes found between studies. For example, one study by Goitein et al. concluded that sleeve gastrectomy and Roux-en-Y are equally safe in the perioperative period [24]. In contrast, a similar study by Robertson et al. showed that perioperative mortality rates were higher for Roux-en-Y than sleeve gastrectomy and gastric banding [25]. Furthermore, a third study by Singhal et al. found that in the GENEVA cohort, there was no significant difference between sleeve gastrectomy, Roux-en-Y, and gastric banding regarding 30-day morbidity and mortality outcomes [26]. More large-scale studies are needed to understand better the actual difference in outcomes between these three surgeries, and our study is a stepping stone towards that objective.

Diabetes is a known risk factor for various intraoperative and postoperative complications of multiple surgeries, and bariatric surgery is no different [27]. The influence of diabetes on postoperative complications and long-term weight loss outcomes following bariatric surgery is a multifaceted area of study. Research suggests that individuals with diabetes undergoing bariatric procedures may face an increased risk of certain postoperative complications, such as surgical site infections, wound healing issues, and gastrointestinal complications, compared to non-diabetes patients [28]. The results of this study are aligned with a large study of the MBSAQIP database, which reported that patients with diabetes were found to have higher perioperative morbidity, serious adverse events, renal events, and cardiac events than non-diabetes patients, regardless of whether they underwent sleeve gastrectomy or Roux-en-Y [29]. Nevertheless, it is already known that bariatric surgery significantly reduces all-cause mortality and diabetes-associated cardiac and renal outcomes in patients with diabetes [30].

Gastric banding is consistently the least-performing bariatric surgery compared to sleeve gastrectomy and Roux-en-Y, according to data from the American Society for Metabolic and Bariatric Surgery (ASMBS) between 2011 and 2021 [31]. One explanation for this is the feared risk of bleeding from gastric erosion that can happen even in the long term with gastric banding [32]. Our study supports that gastric banding was less performed among other surgeries as gastric banding was found to have a 7-fold and 11-fold increase in bleeding risk compared to sleeve gastrectomy and Roux-en-Y, respectively. The bleeding risk was even higher among diabetes patients who underwent gastric banding, precisely three times higher than among non-diabetes patients. Another explanation for the downtrend in gastric banding is the increased overall complication rate, up to 26% [33]. This explanation also supports this study, where the number of cases that underwent gastric banding decreased exponentially between 2015 and 2019. A similar downtrend was observed for Roux-en-Y cases in our study and the ASMBS analysis [31], with a similar reasoning for the decline: worse outcomes when compared to other surgeries. According to a study by Alizadeh et al., Roux-en-Y was associated with significantly worse 30-day morbidity and mortality when compared with sleeve gastrectomy [34]. On the other end of the spectrum, sleeve gastrectomy had an exponential increase in the number of cases in our study, and a similar increase matched this in cases between 2015 and 2019, per the ASMBS data [31]. This is because it is easier to perform and has less morbidity and mortality than other procedures like gastric banding and Roux-en-Y [35]. This supports the current study, where sleeve gastrectomy had significantly less mortality than gastric banding and Roux-en-Y.

This study has limitations that need to be acknowledged. Firstly, this is an observational study, which means there may be inherent biases and, therefore, a lack of randomization, which may impact the results. Secondly, ICD-9 and ICD-10 codes were used when obtaining data from the NIS database, which may be prone to coding errors present within any database. One of the limitations of this analysis is the sample size imbalance in this study. For example, gastric banding cases were disproportionately fewer than sleeve gastrectomy cases. In addition to inherent differences between surgical preferences, one explanation is that gastric banding can be commonly performed in the ambulatory setting and thus not be recorded as much by the hospital-based NIS database [36].

Further, several important cofounders of diabetes, such as the criteria used to diagnose it, its duration, control, and management, are missing. Adjusting those factors might have changed the risk of mortality and morbidity among diabetes patients. Lastly, this study only considers patients in the US, so any results or conclusions may not reflect broader global patterns. Finally, data was collected between 2015 and 2019; hence, outcomes might differ from today’s surgery, which uses easier and safer tools and techniques, and benefits from more considerable experience of surgeons. Despite those limitations, we believe that our analysis provided a recent trend analysis of bariatric surgery in patients with diabetes and confirmed that, despite advances in the management of diabetes, the latter is still associated with high morbidity and mortality.

## 5. Conclusions

In total, we showed that type 2 diabetes is associated with worse postoperative mortality and morbidity in bariatric surgery, regardless of the procedure. Further, Roux-en-Y was associated with higher mortality and morbidity among diabetes patients. Since our data was derived from a retrospective database, causality cannot be implied, and clinical recommendations could not be made. However, special attention should be paid to patients with diabetes to decrease mortality and morbidity. Further research is needed to validate and expand upon the findings, preferably in clinical trials. In addition, research should also be extended to alternatives to surgery, such as pharmacotherapy. Some increasingly studied medications, such as GLP-1 agonists, offer a promising future and potentially a less-invasive alternative to bariatric surgery in the future [37]. A recent study by Sarma et al. reported that, although bariatric surgery had more significant weight reduction in patients, it had a similar effect on glycemic control compared to GLP-1 agonists [38].

## Figures and Tables

**Table 1:** In-hospital outcomes of sleeve gastrectomy in diabetes and non-diabetes patients.

Outcome		Number of Events (%)		Unadjusted OR (95% CI)	Adjusted OR (95% CI)	*p*-Value
		Diabetes	Non-Diabetes			
Mortality	No	133,970 (99.9%)	399,935 (100%)	ref	ref	0.001
	Yes	75 (0.1%)	40 (0.01%)	5.60 (3.81–8.22)	2.07 (1.36–3.16)	
Major bleeding	No	133,370 (99.5%)	397,935 (99.5%)	ref	ref	0.501
	Yes	675 (0.5%)	2040 (0.5%)	0.99 (0.91–1.08)	1.03 (0.94–1.14)	
Atrial fibrillation	No	129,550 (96.6%)	394,185 (98.6%)	ref	ref	0.005
	Yes	4495 (3.4%)	5790 (1.4%)	2.36 (2.27–2.46)	1.07 (1.02–1.11)	
Acute renal failure	No	132,315 (98.7%)	398,610 (99.7%)	ref	ref	<0.001
	Yes	1730 (1.3%)	1365 (0.3%)	3.82 (3.56–4.10)	1.51 (1.39–1.64)	

**Table 2:** In-hospital outcomes of Roux-en-Y in diabetes and non-diabetes patients.

Outcome		Number of Events (%)		Unadjusted OR (95% CI)	Adjusted OR (95% CI)	*p*-Value
		Diabetes	Non-Diabetes			
Mortality	No	472 (98.95%)	2258 (99.39%)	ref	ref	0.355
	Yes	5 (1.05%)	14 (0.61%)	1.71 (0.62–4.85)	1.55 (0.48–5.44)	
Major bleeding	No	472 (98.95%)	2262 (99.58%)	ref	ref	0.989
	Yes	5 (1.05%)	10 (0.42%)	2.49 (0.84–7.37)	2.26 (0.65–7.84)	
Atrial fibrillation	No	443 (92.96%)	2204 (97.02%)	ref	ref	<0.001
	Yes	34 (7.04%)	68 (2.98%)	2.47 (1.61–3.78)	2.24 (1.25–4.02)	
Acute renal failure	No	447 (93.68%)	2130 (93.78%)	ref	ref	<0.001
	Yes	30 (6.32%)	141 (6.22%)	1.01 (0.68–1.53)	1.36 (1.21–1.62)	

**Table 3:** In-hospital outcomes of gastric banding in diabetes and non-diabetes patients.

Outcome		Number of Events (%)		Unadjusted OR (95% CI)	Adjusted OR (95% CI)	*p*-Value
		Diabetes	Non-Diabetes			
Mortality	No	575 (100%)	1610 (99.69%)	ref	ref	-
	Yes	0 (0%)	5 (0.31%)	-	-	
Major bleeding	No	1555 (96.28%)	560 (97.39%)	ref	ref	0.003
	Yes	60 (3.72%)	15 (2.61%)	1.45 (0.81–2.56)	2.94 (1.42–5.88)	
Atrial fibrillation	No	555 (96.52%)	1595 (98.76%)	ref	ref	0.055
	Yes	20 (3.48%)	20 (1.24%)	2.87 (1.54–5.38)	2.23 (0.98–5.04)	
Acute renal failure	No	555 (96.52%)	1580 (97.83%)	ref	ref	0.918
	Yes	20 (3.48%)	35 (2.17%)	1.63 (0.93–2.84)	1.04 (0.49–2.19)	

**Table 4:** In-hospital outcomes of sleeve gastrectomy versus Roux-en-Y among diabetes patients.

Outcome		Sleeve Gastrectomy	Roux-en-Y	*p*-Value
Mortality	N (%)	75 (0.1%)	5 (1.05%)	<0.001
	Adjusted OR (95% CI)	1	8.64 (4.53–16.49)	
Major bleeding	N (%)	675 (0.5%)	5 (1.05%)	0.864
	Adjusted OR (95% CI)	1	1.06 (0.55–2.02)	
Atrial fibrillation	N (%)	4495 (3.4%)	34 (7.04%)	<0.001
	Adjusted OR (95% CI)	1	1.62 (1.48–1.81)	
Acute renal failure	N (%)	1730 (1.3%)	30 (6.32%)	<0.001
	Adjusted OR (95% CI)	1	3.68 (2.92–4.63)	

## Data Availability

Data are available upon a reasonable request from the corresponding author.

## Supplementary Materials

The following supporting information can be downloaded at: https://www.mdpi.com/article/10.3390/jcm13113174/s1. Table S1: Comparison of baseline characteristics of diabetes and non-diabetes patients who underwent sleeve gastrectomy. Table S2: Comparison of baseline characteristics of diabetes and non-diabetes patients who underwent Roux-en-Y. Table S3: Comparison of baseline characteristics of diabetes and non-diabetes patients who underwent gastric banding. Table S4: In-hospital sleeve gastrectomy versus gastric banding outcomes among diabetes patients. Table S5: In-hospital outcomes of Roux-en-Y versus gastric banding among diabetes patients.

## References

[1] Abi Khalil C., Taheri S., Nóbrega C., Rodriguez-López R. (2014). Obesity and Type 2 Diabetes. Molecular Mechanisms Underpinning the Development of Obesity.

[2] Garvey W.T., Mechanick J.I., Brett E.M., Garber A.J., Hurley D.L., Jastreboff A.M., Nadolsky K., Pessah-Pollack R., Plodkowski R., Reviewers of the AACE/ACE Obesity Clinical Practice Guidelines (2016). American Association of Clinical Endocrinologists and American College of Endocrinology Comprehensive Clinical Practice Guidelines for Medical Care of Patients with Obesity. Endocr. Pract..

[3] Eisenberg D., Shikora S.A., Aarts E., Aminian A., Angrisani L., Cohen R.V., De Luca M., Faria S.L., Goodpaster K.P.S., Haddad A. (2022). 2022 American Society for Metabolic and Bariatric Surgery (ASMBS) and International Federation for the Surgery of Obesity and Metabolic Disorders (IFSO): Indications for Metabolic and Bariatric Surgery. Surg. Obes. Relat. Dis..

[4] Abi Khalil C., Roussel R., Mohammedi K., Danchin N., Marre M. (2012). Cause-specific mortality in diabetes: Recent changes in trend mortality. Eur. J. Prev. Cardiol..

[5] Huang D., Refaat M., Mohammedi K., Jayyousi A., Al Suwaidi J., Abi Khalil C. (2017). Macrovascular Complications in Patients with Diabetes and Prediabetes. Biomed. Res. Int..

[6] Rask-Madsen C., King G.L. (2013). Vascular complications of diabetes: Mechanisms of injury and protective factors. Cell Metab..

[7] Yang D.R., Wang M.Y., Zhang C.L., Wang Y. (2024). Endothelial dysfunction in vascular complications of diabetes: A comprehensive review of mechanisms and implications. Front. Endocrinol..

[8] Faselis C., Katsimardou A., Imprialos K., Deligkaris P., Kallistratos M., Dimitriadis K. (2020). Microvascular Complications of Type 2 Diabetes Mellitus. Curr. Vasc. Pharmacol..

[9] Maamoun H., Benameur T., Pintus G., Munusamy S., Agouni A. (2019). Crosstalk Between Oxidative Stress and Endoplasmic Reticulum (ER) Stress in Endothelial Dysfunction and Aberrant Angiogenesis Associated With Diabetes: A Focus on the Protective Roles of Heme Oxygenase (HO)-1. Front. Physiol..

[10] Affinati A.H., Esfandiari N.H., Oral E.A., Kraftson A.T. (2019). Bariatric Surgery in the Treatment of Type 2 Diabetes. Curr. Diab. Rep..

[11] Booth H., Khan O., Prevost T., Reddy M., Dregan A., Charlton J., Ashworth M., Rudisill C., Littlejohns P., Gulliford M.C. (2014). Incidence of type 2 diabetes after bariatric surgery: Population-based matched cohort study. Lancet Diabetes Endocrinol..

[12] Koliaki C., Liatis S., le Roux C.W., Kokkinos A. (2017). The role of bariatric surgery to treat diabetes: Current challenges and perspectives. BMC Endocr. Disord..

[13] Batterham R.L., Cummings D.E. (2016). Mechanisms of Diabetes Improvement Following Bariatric/Metabolic Surgery. Diabetes Care.

[14] Zhang X., Hou A., Cao J., Liu Y., Lou J., Li H., Ma Y., Song Y., Mi W., Liu J. (2022). Association of Diabetes Mellitus With Postoperative Complications and Mortality after Non-Cardiac Surgery: A Meta-Analysis and Systematic Review. Front. Endocrinol..

[15] Steiner C., Elixhauser A., Schnaier J. (2002). The healthcare cost and utilization project: An overview. Eff. Clin. Pract..

[16] NIS (2011). Healthcare Cost and Utilization Project (HCUP).

[17] Buchwald H., Oien D.M. (2013). Metabolic/bariatric surgery worldwide 2011. Obes. Surg..

[18] Billeter A.T., Fischer L., Wekerle A.L., Senft J., Muller-Stich B. (2014). Malabsorption as a Therapeutic Approach in Bariatric Surgery. Viszeralmedizin.

[19] Karmali S., Johnson Stoklossa C., Sharma A., Stadnyk J., Christiansen S., Cottreau D., Birch D.W. (2010). Bariatric surgery: A primer. Can. Fam. Physician.

[20] Salman M.A., El-Ghobary M., Soliman A., El Sherbiny M., Abouelregal T.E., Albitar A., Abdallah A., Mikhail H.M.S., Nafea M.A., Sultan A. (2020). Long-Term Changes in Leptin, Chemerin, and Ghrelin Levels Following Roux-en-Y Gastric Bypass and Laparoscopic Sleeve Gastrectomy. Obes. Surg..

[21] Wang Y., Guo X., Lu X., Mattar S., Kassab G. (2019). Mechanisms of Weight Loss After Sleeve Gastrectomy and Adjustable Gastric Banding: Far More Than Just Restriction. Obesity.

[22] Abdeen G., le Roux C.W. (2016). Mechanism Underlying the Weight Loss and Complications of Roux-en-Y Gastric Bypass. Review. Obes. Surg..

[23] Ozsoy Z., Demir E. (2018). Which Bariatric Procedure Is the Most Popular in the World? A Bibliometric Comparison. Obes. Surg..

[24] Goitein D., Raziel A., Szold A., Sakran N. (2016). Assessment of perioperative complications following primary bariatric surgery according to the Clavien-Dindo classification: Comparison of sleeve gastrectomy and Roux-Y gastric bypass. Surg. Endosc..

[25] Robertson A.G.N., Wiggins T., Robertson F.P., Huppler L., Doleman B., Harrison E.M., Hollyman M., Welbourn R. (2021). Perioperative mortality in bariatric surgery: Meta-analysis. Br. J. Surg..

[26] Singhal R., Cardoso V.R., Wiggins T., Super J., Ludwig C., Gkoutos G.V., Mahawar K., Collaborators G. (2022). 30-day morbidity and mortality of sleeve gastrectomy, Roux-en-Y gastric bypass and one anastomosis gastric bypass: A propensity score-matched analysis of the GENEVA data. Int. J. Obes..

[27] Buchwald H., Avidor Y., Braunwald E., Jensen M.D., Pories W., Fahrbach K., Schoelles K. (2004). Bariatric surgery: A systematic review and meta-analysis. JAMA.

[28] Steele K.E., Prokopowicz G.P., Chang H.Y., Richards T., Clark J.M., Weiner J.P., Bleich S.N., Wu A.W., Segal J.B. (2012). Risk of complications after bariatric surgery among individuals with and without type 2 diabetes mellitus. Surg. Obes. Relat. Dis..

[29] Leonard-Murali S., Nasser H., Ivanics T., Shakaroun D., Genaw J. (2020). Perioperative Outcomes of Roux-en-Y Gastric Bypass and Sleeve Gastrectomy in Patients with Diabetes Mellitus: An Analysis of the Metabolic and Bariatric Surgery Accreditation and Quality Improvement Program (MBSAQIP) Database. Obes. Surg..

[30] Doumouras A.G., Lee Y., Paterson J.M., Gerstein H.C., Shah B.R., Sivapathasundaram B., Tarride J.E., Anvari M., Hong D. (2021). Association Between Bariatric Surgery and Major Adverse Diabetes Outcomes in Patients With Diabetes and Obesity. JAMA Netw. Open.

[31] American Society for Metabolic and Bariatric Surgery Estimate of Bariatric Surgery Numbers, 2011–2021. https://asmbs.org/resources/estimate-of-bariatric-surgery-numbers.

[32] Rao A.D., Ramalingam G. (2006). Exsanguinating hemorrhage following gastric erosion after laparoscopic adjustable gastric banding. Obes. Surg..

[33] Hota P., Caroline D., Gupta S., Agosto O. (2018). Laparoscopic adjustable gastric band erosion with intragastric band migration: A rare but serious complication. Radiol. Case. Rep..

[34] Alizadeh R.F., Li S., Gambhir S., Hinojosa M.W., Smith B.R., Stamos M.J., Nguyen N.T. (2019). Laparoscopic Sleeve Gastrectomy or Laparoscopic Gastric Bypass for Patients with Metabolic Syndrome: An MBSAQIP Analysis. Am. Surg..

[35] Reges O., Greenland P., Dicker D., Leibowitz M., Hoshen M., Gofer I., Rasmussen-Torvik L.J., Balicer R.D. (2018). Association of Bariatric Surgery Using Laparoscopic Banding, Roux-en-Y Gastric Bypass, or Laparoscopic Sleeve Gastrectomy vs. Usual Care Obesity Management with All-Cause Mortality. JAMA.

[36] Sasse K.C., Ganser J.H., Kozar M.D., Watson R.W., Lim D.C., McGinley L., Smith C.J., Bovee V., Beh J. (2009). Outpatient weight loss surgery: Initiating a gastric bypass and gastric banding ambulatory weight loss surgery center. JSLS.

[37] Wang J.Y., Wang Q.W., Yang X.Y., Yang W., Li D.R., Jin J.Y., Zhang H.C., Zhang X.F. (2023). GLP-1 receptor agonists for the treatment of obesity: Role as a promising approach. Front. Endocrinol..

[38] Sarma S., Palcu P. (2022). Weight loss between glucagon-like peptide-1 receptor agonists and bariatric surgery in adults with obesity: A systematic review and meta-analysis. Obesity.

