# Supplementary material for: Diabetes Is Associated with Worse Postoperative Mortality and Morbidity in Bariatric Surgery, Regardless of the Procedure
Source: J Clin Med. 2024 May 28;13(11):3174. doi: 10.3390/jcm13113174 (PMC11173340; doi:10.3390/jcm13113174)
Supplement: Supplementary file 1 [file jcm-13-03174-s001.zip › jcm-2984146-supplementary.pdf]

Supplementary Tables

Supplementary Table S1: Comparison of baseline characteristics of diabetes and non-diabetes patients who underwent sleeve gastrectomy.

		Non-diabetes	***Diabetes***	
Age	Mean (SD)	42 (12)	49 (12)	<0.001
	<55	333260 (83.3%)	88070 (65.7%)	
	55 - 64	50150 (12.5%)	31580 (23.6%)	
	65 - 74	15935 (4%)	13845 (10.3%)	
	≥75	630 (0.2%)	550 (0.4%)	<0.001
Gender	Male	72135 (18%)	38190 (28.5%)	
	Female	327840 (82%)	95855 (71.5%)	<0.001
Race	White	235220 (60.5%)	77350 (59.5%)	
	Black	74810 (19.3%)	26165 (20.1%)	
	Hispanic	59265 (15.3%)	19875 (15.3%)	
	Asian	2480 (0.6%)	1275 (1%)	
	Other minorities	16840 (4.3%)	5415 (4.2%)	0.61
Income	Low	97825 (24.7%)	37910 (28.7%)	
	Low-Mid	99150 (25.1%)	35340 (26.7%)	
	High-Mid	105360 (26.6%)	33270 (25.1%)	
	High	93260 (23.6%)	25775 (19.5%)	<0.001
Comorbidities	Hypertension	133685 (33.4%)	75850 (56.6%)	<0.001
	Smoking	73210 (18.3%)	30790 (23%)	<0.001
	Dyslipidemia	80195 (20.1%)	60040 (44.8%)	<0.001
	PVD	1595 (0.4%)	1305 (1%)	<0.001
	VHD	3160 (0.8%)	1615 (1.2%)	<0.001
	CKD	4610 (1.2%)	8010 (6%)	<0.001
	CAD	5 (0.001%)	40 (0.03%)	<0.001

CAD= Coronary artery disease, CKD= chronic kidney disease, PVD= Peripheral vascular disease, VHD= Valvular heart disease.

<u>Supplementary Table S2:</u> Comparison of baseline characteristics of diabetes and non-diabetes patients who underwent Roux-en-Y.

		Non-diabetes	***Diabetes***	
Age	Mean (SD)	48 (12)	53 (12)	<0.001
	<55	1657 (72.93%)	286 (59.87%)	
	55 - 64	444 (19.55%)	138 (28.91%)	
	65 - 74	111 (4.89%)	35 (7.27%)	
	≥75	60 (2.64%)	19 (3.95%)	<0.001
Gender	Male	1378 (60.67%)	284 (59.57%)	
	Female	894 (39.33%)	193 (40.43%)	0.651
Race	White	1473 (75.5%)	301 (72.64%)	
	Black	320 (16.41%)	71 (17.08%)	
	Hispanic	95 (4.85%)	32 (7.81%)	
	Asian	15 (0.77%)	5 (1.27%)	
	Other minorities	49 (2.47%)	5 (1.21%)	0.097
Income	Low	704 (31.47%)	155 (34.53%)	
	Low-Mid	767 (34.31%)	169 (37.64%)	
	High-Mid	511 (22.86%)	95 (21.1%)	
	High	254 (11.36%)	30 (6.73%)	0.015
Comorbidities	Hypertension	970 (42.69%)	351 (73.71%)	<0.001
	Smoking	1846 (81.26%)	360 (75.43%)	0.005
	Dyslipidemia	479 (21.07%)	197 (41.29%)	<0.001
	PVD	361 (15.89%)	89 (18.56%)	0.137
	VHD	47 (2.06%)	10 (2%)	0.969
	CKD	132 (5.83%)	109 (22.82%)	<0.001
	CAD	166 (7.3%)	94 (19.8%)	<0.001

CAD= Coronary artery disease, CKD= chronic kidney disease, PVD= Peripheral vascular disease, VHD= Valvular heart disease.

<u>Supplementary Table S3:</u> Comparison of baseline characteristics of diabetes and non-diabetes patients who underwent gastric banding.

		Non-diabetes	***Diabetes***	
Age	Mean (SD)	45 (13)	53 (12)	<0.001
	<55	1255 (77.71%)	295 (51.3%)	
	55 - 64	205 (12.69%)	180 (31.3%)	
	65 - 74	135 (8.36%)	90 (15.65%)	
	≥75	20 (1.24%)	10 (1.74%)	<0.001
Gender	Male	395 (24.46%)	180 (31.3%)	
	Female	1220 (75.54%)	395 (68.7%)	0.001
Race	White	895 (57.01%)	315 (55.75%)	
	Black	315 (20.06%)	80 (14.16%)	
	Hispanic	285 (18.15%)	115 (20.35%)	
	Asian	10 (0.64%)	10 (1.77%)	
	Other minorities	65 (4.05%)	45 (7.88%)	<0.001
Income	Low	485 (30.12%)	140 (25.45%)	
	Low-Mid	350 (21.74%)	150 (27.27%)	
	High-Mid	420 (26.09%)	105 (19.09%)	
	High	355 (22.05%)	155 (28.18%)	<0.001
Comorbidities	Hypertension	490 (30.34%)	295 (51.3%)	<0.001
	Smoking	235 (14.55%)	100 (17.39%)	0.106
	Dyslipidemia	290 (17.96%)	220 (38.26%)	<0.001
	PVD	5 (0.31%)	0 (0%)	NA
	VHD	25 (1.55%)	10 (1.74%)	0.754
	CKD	30 (1.86%)	40 (6.96%)	<0.001
	CAD	0 (0%)	0 (0%)	NA

CAD= Coronary artery disease, CKD= chronic kidney disease, PVD= Peripheral vascular disease, VHD= Valvular heart disease, NA= Not applicable.

Supplementary Table S4: In-hospital outcomes of sleeve gastrectomy versus gastric banding among diabetes patients.

Outcome		Sleeve Gastrectomy	Gastric Banding	P-value
**Mortality**	N (%)	75 (0.1%)	0 (0%)	NA
	Adjusted OR (95% CI)	1	NA	
**Bleeding**	N (%)	675 (0.5%)	60 (3.72%)	<0.001
	Adjusted OR (95% CI)	1	7.1 [5.57-9.05]	
**Atrial fibrillation**	N (%)	4495 (3.4%)	20 (3.48%)	0.002
	Adjusted OR (95% CI)	1	0.58 [0.41-0.83]	
**Acute renal failure**	N (%)	1730 (1.3%)	20 (3.48%)	<0.001
	Adjusted OR (95% CI)	1	3.89 [2.9-5.2]	

<u>Supplementary Table S5:</u> In-hospital outcomes of Roux-En-Y versus gastric banding among diabetes patients

Outcome		Roux-en-Y	Gastric Banding	P-value
**Mortality**	N (%)	5 (1.05%)	0 (0%)	NA
	Adjusted OR (95% CI)	1	NA	
**Bleeding**	N (%)	5 (1.05%)	60 (3.72%)	<0.001
	Adjusted OR (95% CI)	1	11.01 (4.89-24.78)	
**Atrial fibrillation**	N (%)	34 (7.04%)	20 (3.48%)	0.056
	Adjusted OR (95% CI)	1	0.59 (0.35-1.01)	
**Acute renal failure**	N (%)	30 (6.32%)	20 (3.48%)	<0.001
	Adjusted OR (95% CI)	1	0.34 (0.22-0.53)	
